# Lymphocyte Activation Markers in Pediatric Kidney Transplant Recipients

**Published:** 2015-09

**Authors:** Fatina I. Fadel, Eman A. Elghoroury, Manal F. Elshamaa, Hafez M. Bazaraa, Doaa M. Salah, Neemat M. A. Kassem, Mona H. Ibrahim, Gamila S. El-Saaid, Soha A. Nasr, Hala M. Koura

**Affiliations:** 1 Department of Pediatric, Faculty of Medicine, Cairo University, Cairo, Egypt;; 2 Department of Clinical & Chemical Pathology, National Research Centre, Cairo, Egypt;; 3 Department of Pediatric, National Research Centre, Cairo, Egypt;; 4 Department of Oncology, Faculty of Medicine, Cairo University, Cairo, Egypt;; 5 Department of Medical Biochemistry, National Research Centre, Cairo, Egypt

**Keywords:** T-lymphocytes, Regulatory, CD25, CD71, Graft Rejection, Children, Kidney Transplantation

## Abstract

**Background and objectives:**

The role of CD4+CD25+ T regulatory cells (Tregs) in immune tolerance in experimental transplantation is very important but the clinical significance of circulating Tregs in the peripheral blood is undetermined. We evaluated the association between the frequency of T cell activation markers CD25 and CD71 and clinical parameters that may affect the level of these T cell markers.

**Methods:**

In 47peditric kidney transplant (KT) recipients and 20 healthy controls, the frequency of T cell activation markers, CD25 and CD71 was measured with flow cytometry after transplantation. Two clinical protocols of induction immunosuppression were used: (*1*) anti-thymocyte globulin (THYMO) group (*n* =29) and Basiliximab (BSX) group (n=10).

**Results:**

The percentage of circulating CD25 after KT was significantly lower than that in the controls. There is no significant difference between KT and the controls s regard to circulating CD71. The percentage of CD25 was significantly increased in children with acute rejection compared with those without acute rejection. Calcineurin inhibitors (CNIs) decreased the frequency of CD25 but mammalian target rapamycin (mTOR) inhibitor did not. The proportion of CD25 significantly decreased in THYMO group during the first year after transplantation.

**Conclusion:**

The frequency of circulating T cell activation marker CD25 in pediatric KT recipients is strongly affected by CNIs, and a high frequency of CD25 is associated with acute rejection during the early posttransplant period. The measurement of T cell activation markers, may become a useful immune monitoring tool after kidney transplantation.

## INTRODUCTION

Maintaining long-term allograft survival is a main role in organ transplantation. Transplant tolerance might be a way, to both reduce rejection and minimize immunosuppressive side effects, therefore improving graft outcome. A better understanding of basic tolerogenic mechanisms (clonal exhaustion, deletion, and immune ignorance) as well as the capital role that T cells play during transplant rejection has resulted in different strategies trying to induce tolerance (1–4); namely, T cell costimulation blockade, mixed chimerism induction, T cell depletion, and tolerance mediated by regulatory T cells (Tregs) (1).

Treg have been described as specialized T lymphocytes that are able to suppress immune responses to self- and nonself-antigens. Multiple populations of Treg have been described (5), including so-called “natural Treg,” a specific subset of T cells coexpressing CD4 and high levels of the IL-2 receptor α chain (CD25) (6) and “inducible CD4+CD25+Foxp3+ regulatory T cells” (iTregs) which develop in the periphery from naive CD4+ T cells after exposure to antigens in a specific cytokine microenvironment, tolerogenic APCs, or immunosuppressive drugs (6).

Subsequently, Treg were also shown to play an important role in the development and maintenance of transplantation tolerance in experimental models (7). In humans, circulating Treg have been shown to inhibit anti-donor immune responses in clinically stable transplant recipients (8–10). In addition to CD4 and CD25, Treg are characterized by the constitutive expression of L-selectin (CD62L) (11), cytotoxic T lymphocyte-associated antigen-4 (12), and glucocorticoid-induced TNF receptor (13), as well as by the intracellular expression of the transcription factor forkhead box P3 (FoxP3) (14). Activated, proliferating lymphocytes are also known to express the molecules transferrin receptor (CD71) on their surface and are thus termed “activation antigens” (15). Ekong et al. (16) reported that using cell surface expression of CD69 and CD71, as well as IFN-γ secretion, we have been able to demonstrate two immunologically distinct phenotypes within a cohort of pediatric liver transplantation recipients. Both active regulation and anergy underlie donor specific hyporesponsiveness. Whether these findings can be used to tailor immunosuppression in liver transplantation recipients remains to be studied in a trial of immunosuppression withdrawal or minimization.

Studies in rodents have shown that Tregs can also play a central role in allograft rejection in transplantation (6). Interestingly, a recent report indicates that Treg can display an upregulated IL-7Rα expression and an inadequate suppressive capacity under specific conditions, *e.g.*, in heart transplant recipients experiencing acute rejection (AR) (15).After transplantation, Tregs are usually found in recipient lymphoid tissue and at the graft site (17, 18). Tregs in both sites can block the initiation of an immune response against the graft. Tregs, which represent approximately 5-10% of peripheral circulating CD4+ T cells in rodents and humans, have also been isolated from the peripheral blood of recipients (19, 20); however, information about the clinical significance of circulating Tregs has to be well studied in pediatric kidney transplantation.

Chronic renal allograft dysfunction is one of the major causes of late renal allograft loss. Many factors have been incriminated to be responsible for chronic allograft dysfunction. Among these are chronic vascular rejection, calcineurin inhibitor (CNI)-related nephrotoxicity, and transplant glomerulopathy (21). In order to limit CNI toxicity, many strategies have been used in maintenance renal transplant patients. Recently, in a randomized prospective study, Stallone *et al* (22) showed that a switch from CNIs to sirolimus was more effective than a 40% reductionin CNI dosage to slow the progression of renal allograft injuries among patients with chronic allograft nephropathy (CAN). Hence, conversion from CNIs to mammalian target rapamycin (mTOR) seems to be a good strategy to prevent chronic allograft dysfunction. However, because of mTOR side effects and its poor tolerance by many patients, an alternative strategy may be the combined use of mycophenolic acid (MMF) and low-dose steroids, without CNIs or mTOR inhibitors (22). The main risk of this strategy may be an increased risk for acute rejection. This retrospective (Case-control) study aimed for assessment of T-cell function (CD25 and CD71) and to evaluate the clinical significance of surface markers CD25 and CD71 in pediatric renal transplantation during posttransplant period when the immunologic response between host and graft is active and strong immunosuppression (IS) is needed to prevent acute rejection.

## METHODS

### Study population and control

Total of 47 consecutive pediatric kidney transplant (KTx) recipients were studied. As controls, 20 healthy controls (HC, 12 males, mean age 10.00 ± 8.80 yr) were included. All transplant recipients received an allograft at the Center of Pediatric Nephrology and Transplantation (CPNT), Children’s Hospital, Cairo University, Egypt. Healthy children were recruited from the Pediatric Clinic of the National Research Centre (NRC). The time elapsed from the time of transplantation to the point of the study is 2.39 ± 0.97 yrs (range 0.5-4.5 yrs). Peripheral blood samples were obtained in HC and KT recipients. In kidney transplantation, blood sample was withdrawn after transplantation. In patients having episodes of acute rejection, the samples were taken during the period of rejection.

### Ethical issues

All of the patients gave informed written consent before participating in the study, which was read and approved by the Ethics Committee of NRC in Egypt.

### Immunosuppressive (IS) regimens

All children received intravenous methylprednisolone (12.5-30 mg per day) on the first month of transplantation, and then oral prednisolone was tapered down to 2.5-7.5 mg/day on the first year of transplantation. We divided the KT patients into 4 groups according to their clinical status and immunosuppression: 1-FK group (n=24) stable patients (defined as patients having a stable graft function (stable serum creatinine over the last 6 months with values less than 150µmol/L, 24-hour proteinuria inferior to 0.5 g/d, no circulating donor-specific antibodies as detected by the Luminex assay (23)); they were receiving a standard a triple-drug maintenance IS, which included (Prednisolone+FK506+MMF), 2-cyclosporine (CsA) group (n=18) defined as patients having a stable graft function (see above) and receiving (Prednisolone +CsA +MMF), 3-FK/ mTOR groups (n=2) defined as patients having a stable graft function (see above) and receiving (Prednisolone +CsA +sirolimus/everolimus), 4-patients with CAN (n=3): defined as children having progressive allograft dysfunction (rise of creatinine and/or proteinuria >0.5 g/d) over recent months, evidence of circulating donor-specific antibodies and a biopsy-proven diagnosis of CAN (on the basis of the Banff ’07 criteria (24)), with detection of C4d in peritubular capillaries.

Initial CsA dose was 10 mg/kg per day by oral route (100-400 mg/day), and target trough levels were ranged from 66 to154 ng/mL in the CsA based immunosuppression. Initial FK506 dose was 0.16 mg/kg per day by oral route (1.5-6 mg/day), and target trough levels were 3-14 ng/mL in the first 3 months and 4.5 ng/mL in the FK506/everolimus group. Initial dose of MMF was 360-1000mg/day, and dose was modified based on adverse effects such as diarrhea or leucopenia. IL-2 receptor blocking antibody (anti-IL-2R Ab, Basiliximab) (Simulect, Novartis Pharmaceuticals, Basel, Switzerland), was given to 10 patients (BSX group) (CsA or FK506 based immunesuppression) 4 hrs before and 3 days after renal transplantation (two 20-mg doses). Anti-thymocyte globulin (ATG) (Thymoglobulin_, Genzyme Transplant, Cambridge, MA) was given to 29 patients (THYMO group) (CsA or FK506 based immunesuppression) from days 0 to 3 (1.5 mg/kg per day, each day), Everolimus was administered 2 mg per day and sirolimus was loaded 6 mg per day and then adjusted dose of 2 mg/day was maintained with target trough level of 5-15 ng/mL.

### Clinical parameters

The potential factors which may affect surface markers CD25 and CD71were included. The number of HLA mismatch, the living donor (related *vs*. unrelated), subclinical acute rejection (AR), episode of cytomegalovirus (CMV) infection), graft function and cyclosporine/FK506 serum concentrations were evaluated. The diagnosis of CMV infection was made when copy number of by real time polymerase chain reaction (RT-PCR) is more than 2,000 copies/mL or with tissue biopsy.

Acute rejection was defined as either borderline/suspicious or acute rejection in patients with stable serum creatinine values at the time of biopsy (25). Delayed graft function (DGF) was defined as the need for dialysis during the first week after transplantation. Graft failure was defined as returning to dialysis.

### Flow cytometric analysis

Fresh blood samples on EDTA (100 ul) with monoclonal antibodies were incubated 20’ in the room temperature in the dark. Samples were lysed with 0.5 ml lysing solution Optilyse C (Beckman Coulter, Brea, CA, USA) 10’ the room temperature in the dark. Lysing reaction was stopped with 1 ml Cell Wash (optimized PBS) (Beckton Dickinson Bioscience, Benelux, Belgium), the pellet suspended in PBS and kept in dark between 2-8°C. Samples were measured on a FC 500 flow cytometer (Beckman Coulter, Brea, CA, USA).

Gating strategy: As described before (26, 27) cells were gated by side scatter and CD4 expression. Subsequently CD 25, CD71 were measured on the cell surfaces. A flow cytometry analysis was performed with at least 100 events in the gate.

### Statistical analysis

Statistical package for social science (SPSS) program version 16.0 was used for analysis of data. Data were summarized as mean ± SD, range or percentage. Histograms and normality plots were used for evaluating the normality of data. For those data with skewed distribution, log transformation was performed before a t-test. Data were valuated between the experimental groups by One-Way Analysis of Variance (ANOVA) or by independent t-test. A *p* value of < 0.05 was considered statistically significant.

## RESULTS

### Cross-sectional Analysis

**Clinical Characteristics.** The demographics of the 47 KTx are summarized in Table 1. In KT recipients, the renal function of the patients was excellent (serum creatinine 0.80 ± 0.24 mg/dL) as a level of assessment at the time of the research. Acute rejection occurred in 17 (29.8%) of 47 patients.

**Table 1:** Baseline and clinical characteristics of kidney transplant recipients

	All Kidney Transplant Recipients (n=47)	FK group (n=24)	CsA group (n=18)	FK/mTORi group (n=2)	*P*-value

General characteristics					
Recipient gender (males/females)	29/18	14/10	13/5	1/1	0.55
Mean recipient age at transplant (years)	9.63 ± 3.33	9.21 ± 3.17	9.75 ± 3.18	12.00 ± 8.49	0.64
Donor age (yr, mean ± SD)	36.49 ± 7.44	37.08 ± 8.42	35.61 ± 6.62	40.00 ± 4.00	0.81
Donor organ source					
Deceased	0	0	0	0	
Living, related	36 (76.60%)	20 (83.33%)	12 (66.67%)	2 (100%)	0.49
Living, unrelated	11 (23.40%)	4 (16.67%)	6 (33.33%)	0	
Number of HLA mismatch	2.28 ± 1.8	2.23 ± 1.51	2.55 ± 0.77	2.50 ± 0.71	0.94
Clinical characteristics					
Serum creatinine at the time of the study (mol/L) (mean ± SD)	0.80 ± 0.24	0.80 ± 0.23	0.72 ± 0.17	0.85 ± 0.21	0.35
AR (%)	17 (29.8%)	12 (50%)	2 (%)	0 (0%)	
CD25%	20.82% ± 9.48	19.16 ± 8.90	22.11 ± 9.46	24.00 ± 1.41	0.32
CD71%	2.84 ± 2.72	2.51 ± 1.85	2.63 ± 3.08	4.50 ± 0.71	0.82
Immunosuppression at the time of the study					
FK506/CsA (%)	29 (61.70%) /18 (38.30%)	24 (100%) / 0 (0%)	0 (0%) /18 (100%)	2 (100%) / 0 (100%)	
Sirolimus/everolimus	2 (4.26%)	0 (0%)	0 (0%)	2 (100%)	
MMF	47 (100%)	24 (100%)	18 (100%)	2 (100%)	
Prednisone	47 (100%)	24 (100%)	18 (100%)	2 (100%)	

FK506, tacrolimus; CsA, cyclosporine; mTOR, mammalian target rapamycin; AR, acute rejection.

For patients with CAN (n=3): the mean recipient age at transplant was10.67 ± 3.10 yrs., while the donor age was37.00 ± 4.36 yrs. The donor organ source was 2 (66.67%), living, related and 1(33.33%) living, unrelated. The number of HLA mismatch was 2.67 ± 0.58. The immunosuppression at the time of the study was FK506, MMF and prednisone (3,100%). No significant difference was found between KTx children and healthy controls as regard to percentage of the other T-cell activation marker, ie, CD71 (2.84% ± 2.72% vs 2.37% ± 0.85%, *P*=0.66). Also when analyzing the various study groups, the percentage of CD71 between the patients groups was not statistically different (*p*=0.82).

**Figure 1:**
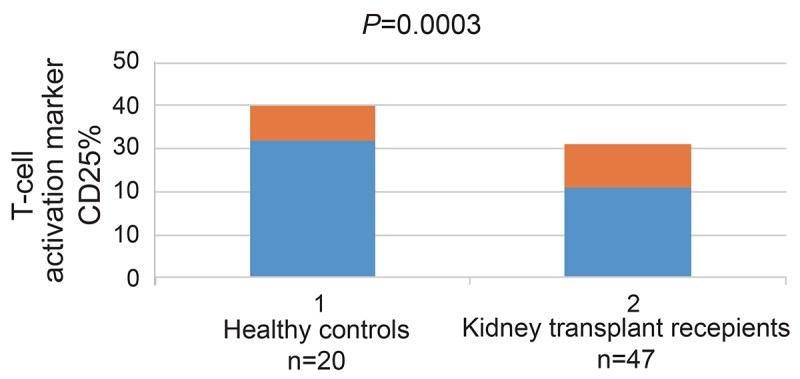
Percentage of CD25 in kidney transplant recipients (mean percentage ± SD). The percentage of CD25 in all KTx was significantly lower as compared with healthy controls (20.82% ± 9.48% *vs* 32.59% ± 7.92%, *p*=0.0003).

**Proportion of CD25and CD71 T Cells.** The percentage of one of the two T-cell activation marker, ie, CD25 was found to be significantly lower in the 47 KTx (mean ± SD, 20.82% ± 9.48%) as compared with the 20 healthy controls (32.59% ± 7.92%, *p*=0.0003) (Figure 1).

### The influence of immunosuppressants on the T cell activation marker, ie, CD25

**Figure 2:**
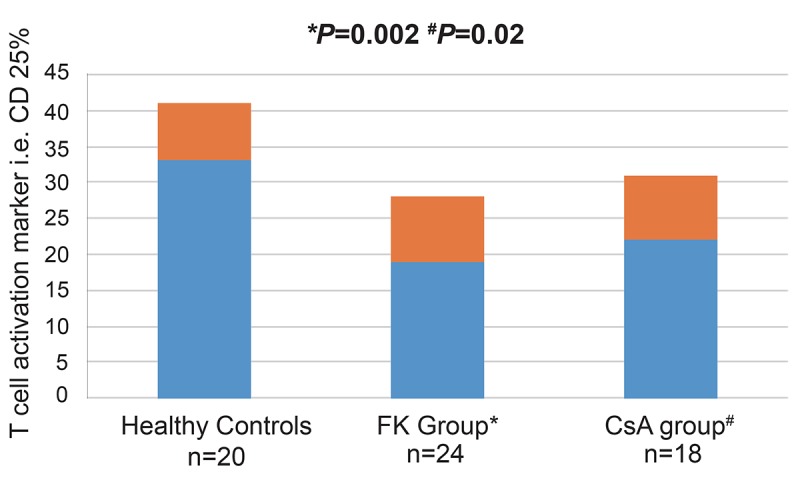
Comparison of the of T cell activation marker CD 25% frequencies based on type of immunosuppressant in kidney transplant recipients. A significant decrease in circulating T cell activation marker CD 25% was observed in the FK506 group (n=24) (19.16 ± 8.90% *vs* 32.59 ± 7.92%, *p*=0.002) and the CsA group (n=18) (22.11 ± 9.46% vs 32.59 ± 7.92%, *p*=0.02) compared with HC. The frequency of CD25 is expressed as a percentage of peripheral blood mononuclear cells (PBMCs).

Figure 2 shows the influence of different immunosuppressants on the T cell activation marker, i.e. CD25. When analyzing the various study groups, the percentage of CD25 between the patients groups was not statistically different; they were found to represent19.16 ± 8.90%in FK group (n=24) and 22.11 ± 9.46% in CsA group (n=18). Of note, the FK group had significantly lower values as compared to controls (*p*=0.002). Similar finding was observed in the CsA group (*p*=0.02). The 2 cases that received the FK/ mTORi IS regimen had 24.00 ± 1.41% of circulating CD25 with 95% confidence interval from -23.68 to 6.51. However, they did not show any significant difference in the circulating CD25 percentage as regards to controls (*p*=0.26). For the 3 patients with CAN the percentage of CD25 was 26.27 ± 6.11% and was also not statistically significant as compared to control (*p*=0.33).

**Figure 3:**
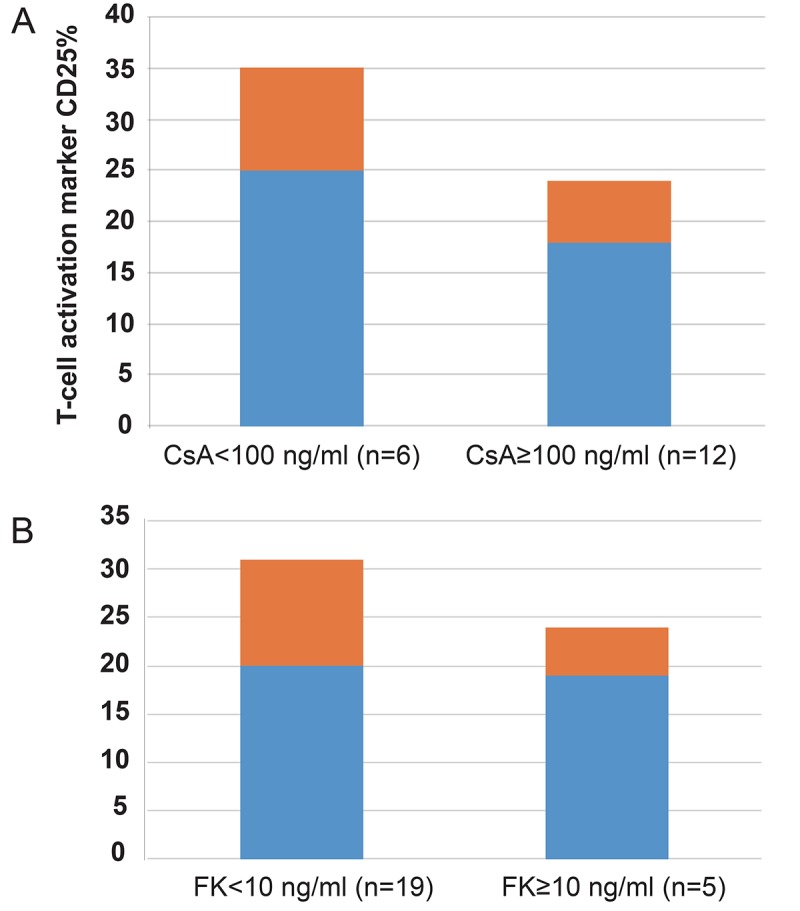
Comparison of circulating T cell activation marker CD 25% frequencies based on immunosuppressive drug levels (A and B) in kidney transplant recipients. Patients with a high blood level of CsA (≥100 ng/mL, A, n=12) showed a lower frequency of circulating CD25% than did patients with low blood levels of CsA (<100 ng/mL, n=6) (17.98 ± 6.24% *vs* 24.68 ± 10.08%, *p*=0.20), but blood levels of FK506 (B) (FK≥10 ng/ml, 19.40 ± 5.43 *vs* FK<10 ng/ml, 19.70 ± 11.03, *p*=0.95) did not affect the T cell activation marker i.e. CD 25%. The frequency of CD25 is expressed as the percentage of peripheral blood mononuclear cells (PBMCs).

We further evaluated whether the blood concentration of the calcineurin inhibitors (CNIs), CsA and FK506, affected the circulating CD25 percentage. The children with CsA high levels (≥100 ng/mL) had insignificantly lower levels of CD25 than those with low CsAlevels (<100 ng/mL) (17.98 ± 6.24% *vs* 24.68 ± 10.08%, *p*=0.20) (Figure 3A); however, the blood levels of FK506 did not affect the level of CD25 (FK≥10 ng/ml, 19.40 ± 5.43 *vs* FK<10 ng/ml, 19.70 ± 11.03, *p*=0.95) (Figure 3B).

### Association between the percentage of CD25 and clinical parameters

**Figure 4:**
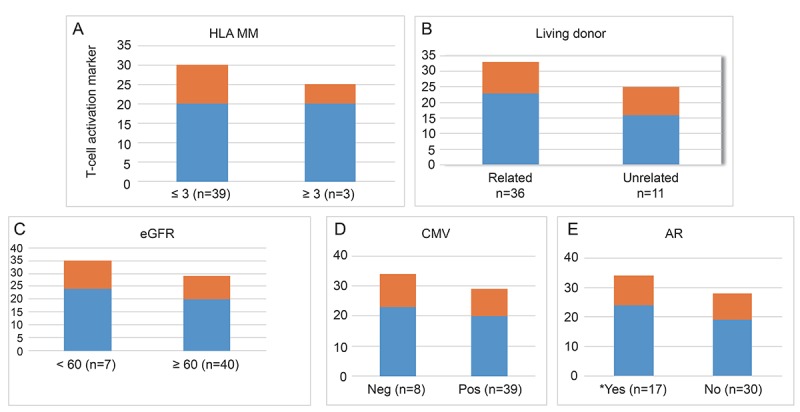
The association between the frequency of T cell activation marker i.e. CD 25% and clinical parameters (A-E) in kidney transplant recipients. The frequency of CD 25 is expressed as a percentage of peripheral blood mononuclear cells (PBMCs). Note that there is no significant association between circulating CD25 and clinical parameters (A-D) except acute rejection (AR) (24.29 ± 9.79%vs19.01 ± 3.83%, *p*=0.04) (E). *denotes *p* values <0.05.

Figure 4 shows the association between the frequency of CD25 and clinical parameters. The number of HLA mismatches, related vs. nonrelated donor, the presence of allograft dysfunction were not correlated with the frequency of CD25 (Fig. 4A-C). Infectious complications such as cytomegalovirus infection also did not lead to any significant changes in the percentage of CD25 (Fig. 4D). Only patients with acute rejection in protocol biopsies had significantly increased frequencies of CD25 compared with those with no detectable rejection (24.29 ± 9.79% vs 19.01 ± 3.83%) (*p*=0.04) (Fig. 4E). So we analyzed these clinical parameters in recipients in whom protocol biopsies were performed (n=17) (Table 2). There were no significant differences between the two groups.

**Table 2:** Comparison of clinical parameters in children with or without acute rejection

	With AR (n=17)	Without AR (n=30)	*P* value

Recipient gender (males/females)	10/7	19/11	0.70
Mean recipient age at transplant (years)	9.37 ± 3.56	10.09 ± 2.95	0.48
Donor age (yr, mean ± SD)	37.00 ± 7.74	36.80 ± 7.52	0.93
Donor organ source			
Living, related	14	22	0.48
Living, unrelated	3	8	
Number of HLA mismatch	2.00 ± 1.65	2.43 ± 0.86	0.26
Immunosuppressants			
FK group	12 (70.58%)	12 (40%)	
CsA group	2 (11.77%)	16 (53.33%)	
CsA/mTORi group	0	2 (6.67%)	
CAN	3 (17.65%)	0	
CD25%	24.29 ± 9.79	19.01 ± 3.83	0.04*
CD71%	2.85 ± 1.79	2.56 ± 2.64	0.70

AR, acute rejection; FK group (Prednisolone+FK506+MMF), CsA group (Prednisolone + CsA + MMF) and FK/mTORi group (Prednisolone + CsA + sirolimus/everolimus), CAN (chronic allograft nephropathy). *P* was significant if <0.05.

### The influence of induction immunosuppressants on the T cell activation marker, ie, CD25

**Figure 5:**
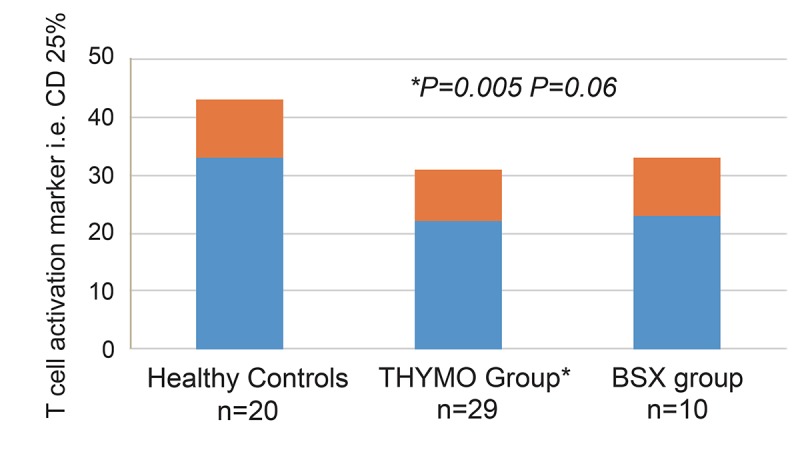
Comparison of the of T cell activation marker CD 25% frequencies based on type of induction immunosuppressant in kidney transplant recipients. A significant decrease in circulating T cell activation marker CD 25% was observed in the THYMO group (n=29) (22.73 ± 9.70% *vs* 32.59 ± 7.92%, *p*=0.005) while CD25 values in BSX group (n=10) was not statistically different as compared to controls (23.42 ± 9.64% vs 32.59 ± 7.92, *p*=0.06). The frequency of CD25 is expressed as a percentage of peripheral blood mononuclear cells (PBMCs).

Figure 5 shows the influence of induction immunosuppressants on the T cell activation marker, i.e. CD25.

Thirty-nine patients were enrolled, all receiving a first kidney transplant.

Two clinical protocols were used: (*1*) THYMO group (*n*=29) and BSX group (n=10). When analyzing the two study groups, the percentage of CD25 between patients groups was not statistically different; they were found to represent 22.73 ± 9.70% in THYMO group and 23.42 ± 9.64% in BSX group (*P*=0.75). Of note, the THYMO group had significantly lower values as compared to controls (*p*=0.005), while CD25 values in BSX group was not statistically different as compared to controls (*p*=0.06).

## DISCUSSION

The aim of this study was to analyze and correlate the T cell activation markers CD25 and CD71 with the clinical status of pediatric renal transplantation (cross-sectional analysis). The results of our study demonstrate that the frequency of circulating CD25 during the posttransplant period in pediatric renal transplant recipients is affected by the type of immunosuppressant treatment and associated with acute rejection. This finding suggests that circulating CD25 expression may be used as a surrogate marker for assessing immune status of renal transplant recipients during the early posttransplant period.

We found that the overall percentages of CD25 T-cells were significantly lower in all KTx to a comparable extent when compared to the controls values. The activation of Tregs depends on the engagement of the T-cell receptor and the presence of IL-2 signaling (28, 29). Therefore, CNIs or anti-CD25 antibody may prevent the generation and maintenance of Tregs by blocking IL-2 signaling or receptor (30). Indeed, CNIs decrease FoxP3 mRNA and protein expression in vitro (31, 32) and reduce the number of peripheral blood Tregs in renal transplant recipients (33). The frequency of circulating Tregs decreased in patients treated with CNI or anti-CD25 antibody, but it was difficult to evaluate the influence of immunosuppressant on circulating CD25% separately because all patients received CNIs . However, a high blood concentration of CsA (≥100 ng/mL) was closely associated with a lower frequency of T cell marker CD25. Therefore, we can at least conclude that number of circulating CD25 is affected by blood level of CsA. Korczak-Kowalska etal.(34) had concluded that CsA therapy resulted in a reduction in the percentage of CD4(+)CD25(+)CTLA-4(+) and CD4(+)CD25(+)Foxp3(+) regulatory T cells after renal transplantation in both groups (uneventful stable courses versus biopsy-proven chronic rejection) compared with patients treated with rapamycin or to healthy donors.

In contrast, mTOR inhibitors selectively expand regulatory T cells *in vivo* (35). Therefore, it is presumable that the patients receiving mTOR inhibitors may have elevated circulating Tregs. In this study, we found that combined treatment of mTOR inhibitors (sirolimus/everolimus) and FK506 did not decrease circulating CD25%. Alhough this result was found for two patients only, this finding may suggest that mTOR inhibitors increase circulating Tregs, but direct effect of mTOR inhibitors on number of circulating Tregs needs to be evaluated. Thus, we cautiously conclude that mTOR inhibitors may compensate the decreased circulating CD25% caused by FK506. Our results on FK/ mTORi IS patients are in accordance with experimental data, indicating that sirolimus relatively spares Treg (as compared with other IS medications) (36-40). Recent studies suggest that mammalian target of rapamycin inhibitors such as sirolimus, by contrast to CNIs, do not interfere with the suppressive capacity of Treg (41) and can induce *de novo* expression of FoxP3 in alloantigen-specific T cells (42) and an increase in the number of functional Treg in KTx (43), indicating that mammalian target of rapamycin inhibitors may favor Treg survival and function in the context of transplantation.

It is widely accepted that Tregs play a pivotal role in tolerance induction (17). Therefore, an increase in circulating Tregs may be beneficial to the grafted kidney in terms of immune tolerance. The results of our study revealed that the frequency of circulating CD25 is significantly reduced by strong immunosuppressants such as CsA or FK506, and that a high blood concentration of CsA further reduced the frequency of circulating T cell activation marker i.e. CD25%. This finding suggests that high doses of CNIs at the time of transplantation prevent the development of Tregs (44). Thus, treatment with CsA or FK506 in the early posttransplant period should be used cautiously and high doses of CNIs should be avoided because it may inhibit the development of immune tolerance. Further evaluation of the long-term effect of CNIs on circulating Tregs and the optimal frequency of circulating Tregs during the early transplant period in kidney transplantation is needed.

In this study the percentage of T cell marker CD 71 is not significantly different from that of controls. This result further confirms that CD4^+^CD25^high^ T cells in human are heterogenous in terms of phenotype and function (27). The finding of nearly normal values of CD71 may indicate that transferrin receptor is not always decreased after transplantation.

To evaluate whether clinical parameters affect the frequency of T cell activation marker i.e. CD25 we evaluated the immunologic factors (the number of HLA mismatches, related vs. nonrelated donor, presence of acute rejection), infectious factors (cytomegalovirus) and allograft function (presence of allograft dysfunction). The results of our study revealed that the frequency of circulating CD25 has no significant association with these clinical parameters except acute rejection which showed high percentage of T cell activation marker i.e.CD25. During acute rejection of kidney allografts, an augmented FOXP3 gene expression as well as increased CD4^+^CD25^+^FOXP3^+^ and other cell populations are observed in graft biopsies. However, it is not clear whether Tregs migrate into the graft and are retained there to suppress the inflammatory process, or whether they are directly associated with more complex mechanisms to induce immune tolerance. There are in vitro and in vivo evidences showing that T cells are able to suppress antigen-specific responses via direct cell-to-cell contact, secrete anti-inflammatory cytokines such as TGF-β and IL-10, and inhibit the generation of memory T cells, among others (45). Overall, these results suggest that the level of activation of T cells is different depending on the degree of allograft acceptance.

Published reports on the use of lymphocyte activation markers in human solid organ transplantation have mostly been limited to cell surface expression of these markers on resting lymphocytes (46, 47), and have described a strong association between biopsy proven rejection and CD 69 expression on CD4+ and CD8+ T cells in peripheral blood. Similarly, immunohistochemical analysis of rejection biopsy specimens has shown that a high proportion of graft-infiltrating T cells express CD 69 (48, 49, 50). Vallotton *et al.* (51) reported that T activated cells were found to be expanded in the circulation of most KTx, with the highest values observed in patients with CHR. Therefore, the frequency of circulating T cell marker CD25 may be used as a surrogate marker for predicting subclinical acute rejection during the early posttransplant period. However, not all reports have shown an association between lymphocyte activation markers and rejection (52), perhaps because of differences in the duration from transplantation between the groups studied, as well as differences in the severity of rejection episodes.

In this study, we found that in THYMO group the proportion of CD25 decreased after transplantation. This reduction of Treg after thymoglobulin induction in clinical kidney transplantation has been previously described (51, 53). Our results indicate that despite standard induction and maintenance IS, there was a rapid reduction of Treg after transplantation, which was persistent over time. This observation suggests that monitoring Treg after kidney transplantation could be of interest in assessing the alloimmune reaction of the recipient vis-a’-vis the graft in the early post-transplant period.

In summary, monitoring of circulating T cell activation marker i.e. CD25 in peripheral blood is helpful for evaluating the immune status of kidney transplant recipients during the early posttransplant period. We demonstrated that expression of CD 25 on peripheral blood T cells correlates closely with the presence of acute graft rejection in renal allograft pediatric recipients. Measurement of this surface marker may provide a rapid, noninvasive, and accurate means by which graft rejection can be identified. The data presented here provide the scientific basis for implementing the monitoring of T cell activation marker CD25 in large prospective clinical studies. Prospective monitoring of T cell activation marker CD25 (*e.g.*, in addition to *de novo* anti-HLA antibodies) may be useful in the diagnosis of graft rejection as well as during the so-called IS minimization strategies.
